# Multidisciplinary treatments for hepatocellular carcinoma with major portal vein tumor thrombus

**DOI:** 10.1007/s00595-013-0585-6

**Published:** 2013-04-17

**Authors:** Satoshi Katagiri, Masakazu Yamamoto

**Affiliations:** Department of Surgery, Institute of Gastroenterology, Tokyo Women’s Medical University, 8-1 Kawada-cho, Shinjuku-ku, Tokyo 162-8666 Japan

**Keywords:** Hepatocellular carcinoma, Surgical treatment, Transcatheter arterial chemoembolization, Hepatic arterial infusion, Radiation, Multimodality treatment

## Abstract

In recent years, various treatment options have become available for patients with hepatocellular carcinoma (HCC) according to the degree of background liver damage, tumor diameter and other factors associated with disease progression. Therapy has also shifted toward evidence-based treatment. Policies for the management of HCC with portal vein tumor thrombus, which has been considered an intractable condition, have not been established. Surgical resection was previously positioned as the treatment of choice, but the outcomes after resection alone were found to be disappointing. At present, multiple interdisciplinary treatments, combining resection with intra-arterial chemotherapy, radiotherapy, systemic chemotherapy and/or immunotherapy, are used on a trial-and-error basis since no standard regimens have been developed. Clinical trials of surgery combined with transarterial chemoembolization, hepatic arterial infusion of chemotherapy and radiation have obtained improved 5-year survival rates of 21.5–56 %. The safety of surgical resection in HCC with major portal vein tumor thrombus has improved, but the optimal type(s) and timing of auxiliary therapy to use in combination with resection remain to be defined.

## Introduction

Hepatocellular carcinoma (HCC) is one of the most common malignant tumors worldwide [1]. The treatment strategies include hepatic resection, transcatheter arterial chemoembolization (TACE), percutaneous ethanol injection (PEI), microwave coagulation therapy (MCT), radiofrequency ablation (RFA), liver transplantation and systemic chemotherapy with sorafenib, an oral multikinase inhibitor [2–8]. In patients with HCC, these treatments are particularly effective for the local control of intrahepatic and extrahepatic lesions, contributing to improved cumulative survival. The mortality and morbidity rates after hepatic resection have improved in recent years because of sophisticated surgical techniques and better perioperative management [9]. However, the outcomes of patients who have HCC with portal vein tumor thrombus (PVTT) remain poor, with a mortality rate much higher than that of HCC without PVTT. The natural history of untreated nonsurgical HCC with PVTT was reported to be associated with a median survival time of 2.7 months [10]. The optimal treatment for HCC with PVTT has not been established, and only a few randomized controlled trails have been conducted. This review summarizes the current knowledge regarding multiple interdisciplinary treatments for HCC with major PVTT.

## Clinical features according to the macroscopic and microscopic classifications of HCC with PVTT

The Liver Cancer Study Group of Japan proposed a macroscopic classification for HCC with PVTT in the General Rules for the Clinical and Pathological Study of Primary Liver Cancer [11]. This classification is useful, because it is based on the clinical characteristics, imaging findings, pathological findings and surgical outcomes.

PVTT is classified into five grades, Vp0–Vp4. Each grade is defined as follows: Vp0, no tumor thrombus in the portal vein; Vp1, presence of a tumor thrombus distal to, but not in, the second-order branches of the portal vein; Vp2, presence of a tumor thrombus in the second-order branches of the portal vein; Vp3, presence of a tumor thrombus in the first-order branches of the portal vein; and Vp4, presence of a tumor thrombus in the main trunk of the portal vein or a portal vein branch contralateral to the primarily involved lobe (or both).

**Fig. 1:**
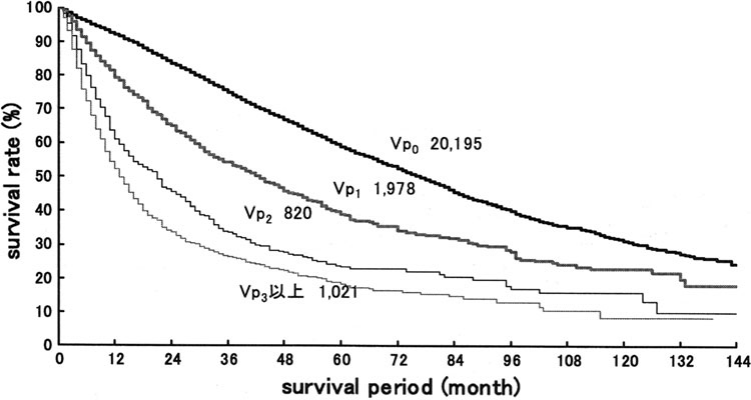
Eighteenth follow-up survey of primary liver cancer conducted in 25,066 patients with HCC treated by hepatic resection between 1994 and 2005. *Vp0* no tumor thrombus; *Vp1* tumor thrombus distal to the second-order branches of the portal vein, but not involving the second-order branches; *Vp2* tumor thrombus in the second-order branches of the portal vein; *Vp3* tumor thrombus in the first-order branches of the portal vein and/or the main trunk of the portal vein and/or contralateral portal vein branch to the primarily involved

**Fig. 2:**
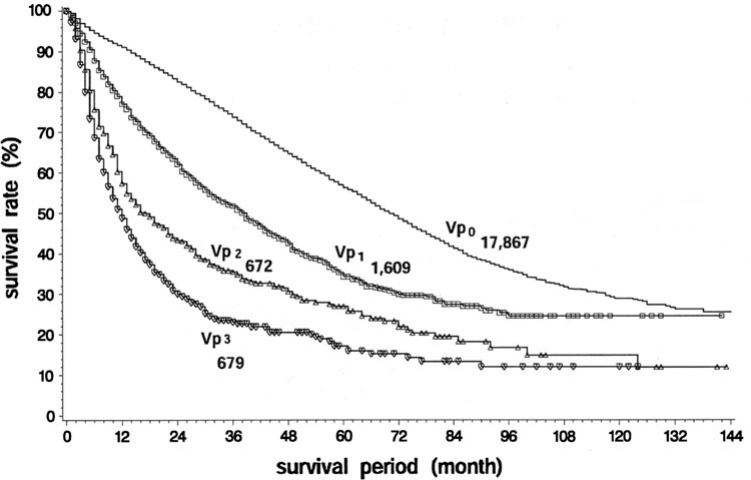
Fifteenth follow-up survey of primary liver cancer conducted in 21,711 patients with HCC treated by hepatic resection between 1988 and 1999. *Vp0* no tumor thrombus; *Vp1* tumor thrombus distal to the second-order branches of the portal vein, but not involving the second-order branches; *Vp2* tumor thrombus in the second-order branches of the portal vein; *Vp3* tumor thrombus in the first-order branches of the portal vein and/or the main trunk of the portal vein and/or contralateral portal vein branch to the primarily involved

The Liver Cancer Study Group of Japan has reported the results of the 18th follow-up survey of primary liver cancer in Japan [12]. Of 17,455 patients with HCC, 86.9, 3.0, 2.8, 3.9 and 3.3 % had Vp0, Vp1, Vp2, Vp3 and Vp4, respectively, on the basis of imaging studies. Of 5,368 patients with HCC examined for the microscopic findings of surgical or biopsy specimens, 74.0, 19.0, 3.1, 2.6 and 1.4 % had grade Vp0, Vp1, Vp2, Vp3 and Vp4 disease, respectively. Of 25,066 patients with HCC treated by hepatic resection between 1994 and 2005, the 5-year cumulative survival rates were 59.0, 39.1, 23.3 and 18.3 % in patients with Vp0, Vp1, Vp2, and Vp3 or Vp4 grade disease, respectively (Fig. 1). In the earlier 15th follow-up survey of primary liver cancer conducted in 21,711 patients with HCC treated by hepatic resection between 1988 and 1999, the 5-year cumulative survival rates were 56.5, 34.4, 27.0 and 17.3 % in patients with Vp0, Vp1, Vp2 and Vp3 or Vp4 grade disease, respectively (Fig. 2) [13]. The results of these surveys indicate that the recent surgical outcomes in patients with Vp2 and Vp3 or Vp4 disease have not improved significantly in Japan during the last 6 years.

## Hepatectomy

In the 1980s, surgical resection was indicated only for patients with a tumor thrombus in a first-order branch of the portal vein, not involving the confluence of the right and left portal veins [14, 15]. Beginning in the 1990s, surgical resection of a tumor thrombus extending to the main portal trunk was reported by Kumada et al. and Yamaoka et al. [16, 17]. The technique was gradually refined and standardized to become the currently used procedure. The results of surgical resection reported in 2000, and subsequently in patients with a PVTT classification of Vp3 and Vp4, are shown in Table 1 [12, 18–26]. These studies basically summarized the outcomes of surgical therapy as the initial treatment after the diagnosis of PVTT, and studies in which patients received pretreatment are not included. The mortality ranged from 0 to 11.5 %, and was less than 5 % in seven of the nine studies in which mortality data were reported. The 5-year survival rates ranged from 0 to 39 %. Although these outcomes were not necessarily the results of resection alone, Wu et al. [18], Inoue et al. [23] and Ban et al. [25] obtained higher 5-year survival rates exceeding 20 %. The Liver Cancer Study Group of Japan reported survival rates of 18.3 % at 5 years and 8.4 % at 10 years in the 18th follow-up survey of primary liver cancer in Japan, encompassing 1,021 patients who underwent Vp3 or Vp4 resection.

**Table 1:** Hepatectomy in patients with HCC and major PVTT

First author	Year	No.	Clarification of PVTT	Mortality	5-year survival rate	Median survival time
Wu [18]	2000	15	Vp 4	0 %	26.4 %	NA
Poon [19]	2003	20	Vp 3–4	5.7 %	13.3 %	6.0 mo
Ikai [20]	2006	78	Vp 3–4	3.8 %	10.9 %	8.9 mo
Treut [21]	2006	26	Vp 3–4 (or Vv)	11.5 %	13.0 %	9.0 mo
Chen [22]	2006	152	Vp 4	2.6 %	0 %	10.1 mo
Inoue [23]	2009	20	Vp 4	0 %	39.0 %	NA
Kondo [24]	2009	5	Vp 4	0 %	0 %	8.0 mo
Ban [25]	2009	45	Vp 3–4	0 %	22.4 %	20.0 mo
Shi [26]	2010	247	Vp 3 (169)	0.6 %	17.7 % (3-year)	15.0 mo
			Vp 4 (78)	0 %	3.6 % (3-year)	10.0 mo
Ikai [12]	2010	1,021	Vp 3–4	NA	18.3 %	NA

*Vp3* tumor thrombus in first-order branches of the portal vein, *Vp4* tumor thrombus in the main trunk of the portal vein and/or contralateral portal vein branch to the primarily involved lobe, *NA* not available, *mo* months, *Vv* tumor thrombus in the hepatic vein

## Transcatheter arterial chemoembolization and hepatic arterial infusion chemotherapy

TACE is a key treatment for patients with unresectable HCC. However, TACE had previously been contraindicated in patients with PVTT involving the main trunk or a first-order left or right branch of the portal vein [27, 28]. In 1997, Lee et al. [29] reported that TACE could be safely performed even in HCC associated with occlusion of the main trunk of the portal vein owing to the presence of collateral circulation. Table 2 shows the results of TACE and hepatic arterial infusion (HAI) chemotherapy in patients who had HCC with high-grade PVTT, reported after 2000 [30–35]. The majority of studies used HAI with a combination of cisplatin and 5-fluorouracil, rather than TACE or transcatheter arterial embolization (TAE). The best results were obtained by Ando et al., who treated 48 patients with Vp2 to Vp4 PVTT by HAI with cisplatin plus 5-fluorouracil. The 5-year overall survival rate was 11.0 %, and the median survival time was 10.2 months in that study [31]. Many of the other studies reported overall survival of up to 3 years, but the long-term outcomes remain largely unclear. The median survival time ranged from 3.5 to 9.5 months, and fell short of 1 year. Although the background characteristics of the study groups differed, there was an overall trend toward shorter survival of patients who underwent HAI compared with studies of patients who underwent surgical resection.

**Table 2:** Transcatheter arterial chemoembolization and hepatic arterial infusion chemotherapy in patients with HCC and major PVTT

First author	Year	No.	Classification of PVTT	Treatment		Survival rate	Median survival time
Itamoto [30]	2002	7	Vp 3–4	HAI	CDDP + 5-FU	NA	7.5 mo
Ando [31]	2002	48	Vp 2–4	HAI	CDDP + 5-FU	11.0 % (5-year)	10.2 mo
Izaki [32]	2004	15	Vp 2–4	GIA-TAE (10)		13.3 % (3-year)	9.5 mo
				GIA-TAE + RT (5)		0 % (3-year)	7.1 mo
Georgiades [33]	2005	32	Vp 3–4	TACE		25.0 % (1-year)	9.5 mo
Akiyama [34]	2008	23	Vp 3–4	HAI	CDDP + 5FU (10)	NA	3.5 mo
					Control (13)	NA	2.2 mo
Kondo [35]	2010	24	Vp 1–4	HAI	CDDP powder	16 % (2-year)	7.0 mo

*Vp1* tumor thrombus distal to the second-order branches of the portal vein, but not involving the second-order branches; *Vp2* tumor thrombus in the second-order branches of the portal vein; *Vp3* tumor thrombus in the first-order branches of the portal vein; *Vp4* tumor thrombus in the main trunk of the portal vein and/or contralateral portal vein branch to the primarily involved lobe; *HAI* hepatic arterial infusion; *CDDP* cisplatin; *5-FU* 5-fluorouracil; *NA* not available; mo, months; *GIA-TAE* transcatheter arterial embolization with gelatin sponge immersed in an anticancer agent; *RT* radiation therapy; *TACE* transcatheter arterial chemoembolization

## Radiotherapy and ablation therapy

The results of a study in which radiotherapy was used to treat PVTT were reported by Chen et al. [36] in 1994. Ten patients were treated, and the response rate was 100 %. Pilot and other studies followed, and many reports appeared after 2000. The studies of radiotherapy and ablation therapy that were reported in 2005 and subsequently are shown in Table 3 [37–45].

**Table 3:** Radiotherapy and ablation therapy in patients with HCC and major PVTT

First author	year	No.	Classification of PVTT	Treatment	Survival rate	Median survival time
Hata [37]	2005	12	Vp 3–4	Proton beam therapy (50–72 Gv)	24 % (5-year)	11 mo (CR + PR)
Nakagawa [38]	2005	52	Vp 2–4	3D-CRT (39–60 Gy)	5.1 % (5-year)	NA
Zeng [39]	2005	44	Vp l–4, Vv3	External beam radiation (36–60 Gy)	34.8 % (1-year)	8.0 mo
Kim [40]	2005	59	Vp 3–4	3D-CRT (39–70.2 Gy)	20.7 % (2-year)	10.7 mo (CR + P)
Lin [41] [RCT]	2006	43	Vp 3–4	Stereotactic radiotherapy (22)	NA	6.0 mo
				3D-CRT (21)	NA	6.7 mo
Zhang [42]	2008	10	Vp 3	125-iodine seed implantation for PVTT	NA	NA
Shirai [42]	2009	26	Vp 3–4	3D-CRT using SPECT	30 % (2-year)	10.3 mo
Giorgio [44]	2009	13	Vp 4	Percutaneous RFA	77 % (3-year)	NA
Zheng [45]	2009	108	Vp 3–4	Percutaneous laser ablation	22.38 % (3-year)	NA

*Vp1* tumor thrombus distal to the second-order branches of the portal vein, but not involving the second-order branches; *Vp2* tumor thrombus in the second-order branches of the portal vein; *Vp3* tumor thrombus in the first-order branches of the portal vein; *Vp4* tumor thrombus in the main trunk of the portal vein and/or contralateral portal vein branch to the primarily involved lobe; *mo* months; *CR* complete response; *PR* partial response; *3D-CRT* three-dimensional conformal radiotherapy; *NA* not available; *Vv* tumor thrombus in the hepatic vein; *RCT* randomized control study; *SPECT* single photon emission computed tomography; *RFA* radiofrequency ablation therapy

Three-dimensional conformal radiotherapy (3D-CRT) was used in four studies [38, 40, 41, 43], and photon beam therapy [37], iodine-125 seed implantation [42], percutaneous radiofrequency ablation [44] and percutaneous laser ablation [45] were used in one study each. The overall survival rates at 5 years were reported only by Hata et al. [37] and Nakagawa et al. [38] and were 24 and 5.1 %, respectively. The longest median survival times were obtained by Hata et al. (11 months) [37], Lin et al. (10.7 months) [41] and Shirai et al. (10.3 months) [43], but were all less than 1 year. Zeng et al. [39] showed that radiotherapy combined with supportive care, TACE or hepatic resection significantly improved the outcomes in a study of 158 patients with HCC with portal vein and/or inferior vena cava tumor thrombus. In a multivariate analysis, the presence or absence of radiotherapy was clearly shown to be a significant determinant of survival. This study provided evidence supporting the therapeutic effectiveness of multimodality treatment.

## Nonsurgical multimodality treatment

Multimodality treatment is clearly essential for the management of HCC and is of particularly high value in cases of HCC with PVTT. Table 4 lists the studies of nonsurgical interdisciplinary treatment in patients with HCC and PVTT [46–56]. The multimodality treatment consisted of HAI plus interferon in five studies [46, 47, 50, 52, 54], HAI with enteric-coated tegafur/uracil (UFT) in one study [53], a combination of 3D-CRT and thalidomide in one study [51], 3D-CRT after TACE in one study [49], radiotherapy after TACE in one study [48], HAI and 3D-CRT combined with interferon in one study [54], TACE after percutaneous transhepatic portal vein stenting (PTPVS) in one study [55], TACE and 3D-CRT after PTPVS in one study [56] and iodine-125 seed strands after PTPVS in one study [56]. The 5-year overall survival rate was 16.4 % in the study of HAI plus interferon reported by Ota et al. [50]. Among the reports on the various types of multimodality treatment, the longest median survival times were obtained by Ota et al. (11.8 months) [50] and in the HAI plus UFT study by Ishikawa et al. (14.7 months) [53]. The investigation by Ota et al. [50] had a relatively large study group and good long-term survival, and is thus considered a valuable clinical trial.

**Table 4:** Nonsurgical interdisciplinary treatment in patients with HCC and major PVTT

First author	Year	No.	Classification of PVTT	Multimodality treatment	Survival rate	Median survival time
Kaneko [46]	2001	8	Vp 3–4	HAI: CDDP,5-FU, MTX + IFM-α + Leu	15 % (2-year)	11 mo (CR + PR)
Sakon [47]	2002	8	Vp 3–4	HAI: 5-FU + IFN-α	NA	NA
Ishikura [48]	2002	20	Vp 3–4	TACE → RT (50 Gy)	25 % (1-year)	5.3 mo
Yamada [49]	2003	19	Vp 3–4	TACE → 3D-CRT	10.2 % (2-year)	7.0 mo
Ota [50]	2005	55	Vp 3–4	HAI: 5-FU + IFN-α	164 % (5-year)	11.8 mo
Hsu [51]	2006	20	Vp 3–4	3D-CRT + thalidomide	0 % (5-year)	NA
Obi [52]	2006	116	Vp 3–4	HAI: 5-FU + IEN-α	18 % (2-year)	6.9 mo
Ishikawa [53]	2007	10	Vp 3–4	HAI: etoposide, carboplatin, epirubicin, 5FU → UFT-E	20 % (2-year)	14.7 mo
Kitamura [54]	2009	32	Vp 3–4	HAI: 5-FU + IFN-α + 3D-CRT (16)	NA	7.5 mo
				HAI: 5-FU + IFN-α (16)	NA	7.9 mo
Zhang [55]	2009	45	Vp 4	PTPVS-TACE → 3D-CRT (16)	32.5 % (360-day)	NA
				PTPVA-TACE (29)	6.9 % (360-day)	NA
Luo [56]	2010	32	Vp 3–4	125-iodine seed strand + Stent	39.3 % (360-day)	8.4 mo

*Vp3* tumor thrombus in first-order branches of the portal vein, *Vp4* tumor thrombus in the main trunk of the portal vein and/or contralateral portal vein branch to the primarily involved lobe, *HAI* hepatic artery infusion chemotherapy, *CDDP* cisplatin, *5-FU* 5-fluorouracil, *MTX* methotrexate, *IFN-α* interferon-α, *Leu* leucovorin, *mo* months, *CR* complete response, *PR* partial response, *NA* not available, *TACE* transcatheter arterial chemoembolization, *RT* radiotherapy, *3D-CRT* three-dimensional conformal radiotherapy, *UFT-E* enteric-coated tegafur/uracil, *PTPVS* percutaneous transhepatic portal vein stenting

## Surgical multimodality treatment

Although direct comparisons of the outcomes of treatment are precluded by the differences in the patients’ background characteristics, hepatectomy appears to provide better outcomes than TACE, TAI, radiotherapy, ablation therapy and nonsurgical multimodality treatment. Table 5 presents the studies of hepatectomy-based interdisciplinary treatment for HCC with PVTT [57–64]. The main treatments used were hepatic resection after TACE in one study; TACE, HAI, and portal vein infusion (PVI) chemotherapy after hepatic resection in three studies; preoperative intravenous chemotherapy with doxorubicin, cisplatin and 5-fluorouracil plus subcutaneous interferon-α (PIAF) or yttrium-90 plus doxorubicin in one study; post-operative percutaneous isolated hepatic perfusion (PIHP) in one study; interferon with 5-fluorouracil after hepatic resection in one study; and hepatic resection after radiotherapy in one study. Hepatic resection was performed before the other treatments in five studies. The 5-year survival rates were reported for all but one study, and were good, ranging from 21.5 to 56 %. The highest rate of 56 % was obtained by Lau et al. [60] in a small study of only seven patients. However, these results were very encouraging. The median survival time after hepatectomy-based multimodality treatment ranged from 13.0 to 22.1 months, suggesting that interdisciplinary therapy contributed to improved long-term survival. In a controlled trial by Peng et al., 126 patients with HCC and PVTT were randomly assigned to hepatectomy alone (control group) or hepatectomy followed by TACE (TACE group). The median survival time was 13 months in the TACE group and 9 months in the control group. The estimated survival rates at 5 years were also better in the TACE group (21.5 %) than in the control group (8.5 %). This randomized controlled study of multimodality treatment is considered to be a key clinical trial. The available evidence indicates that hepatectomy-based interdisciplinary therapy is effective and should be explored in further trials.

**Table 5:** Surgical interdisciplinary treatment it patients with HCC and major PVTT

First author	Year	No.	Classification of PVTT	Multimodality treatment and hepatic reaction	5-year survival rate	Median survival time
Minagawa [57]	2001	45	Vp 2–4	Pre-TACE → Hr (18)	42 %	NA
				TACE or HAI (27)	0 %	NA
Fan [58]	2001	147	Vp 3–4	Conservative (l8)	0 %	2.0 mo
				HAL and/or Post-HAI, PVI (18)	0 %	5.0 mo
				Hr (79)	16.6 %	12.0 mo
				Hr → Post-TACE or HAI and/or PVI (32)	26.8	16.0 mo
Fukuda [59]	2002	19	Vp 3–4 or Vv or B	Hr → Post-HAI or TACE etc.	36.3 %	22.1 mo
Lau [60]	2004	7	Vp 4	Pre-PIAF or yttrium 90 + Dox → Hr	56 %	NA
Ku [61]	2004	17	Vp 1–4	Hr → Post-PIHP	40 %	NA
Nagano [62]	2007	30	Vp 4	Hr → Post-IFN/5-FU	21.4 % (3-year)	9.5 mo
Kamiyama [63]	2007	43	Vp 3–4	Pre-RT → Hr (15)	34.8 %	19.6 mo
				Hr (28)	13.1 %	9.1 mo
Peng [64] [RCT]	2009	126	Vp 3–4	Hr (53)	8.5	9.0 mo
				Hr → Post-TACE (51)	21.5 %	13.0 mo

*Vp1* tumor thrombus in distal to the second-order branches of the portal vein, but not of the second-order branches; *Vp2* tumor thrombus in the second-order branches of the portal vein; *Vp3* tumor thrombus in the first-order branches of the portal vein; *Vp4* tumor thrombus in the main trunk of the portal vein and/or contralateral portal vein branch to the primarily involved lobe; *TACE* transcatheter arterial chemoembolization; *Hr* hepatic resection; *NA* not available; *HAI* hepatic artery infusion chemotherapy; *HAL* hepatic artery ligation; *PVI* portal vein infusion chemotherapy; *Vv* tumor thrombus in the hepatic vein; *B* tumor thrombus in the bile duct; *PIAF* doxorubicin, cisplatin, 5-fluorouracil iv + interferon-α sc; *Dox* doxorubicin; *PIHP* percutaneous isolated hepatic perfusion; *IFN* interferon-α; *5-FU* 5-fluorouracil; *RT* radiotherapy; *RCT* randomized control study

## Conclusions

No curative treatment is currently available for HCC with major PVTT. However, a growing body of evidence suggests that hepatectomy- and thrombectomy-based multiple interdisciplinary treatments are effective options. The details and optimal timing of auxiliary treatments combined with hepatectomy and thrombectomy in patients with HCC and PVTT remain an important topic for future research. Future recommendations must be based on clear evidence from large, well-controlled clinical trials.
